# Exploring the Potential of Bambara Groundnut Flour as an Alternative for Diabetic and Obese Patients in the USA: A Comprehensive Review

**DOI:** 10.7759/cureus.78258

**Published:** 2025-01-30

**Authors:** Oluwasanmi M Odeniran

**Affiliations:** 1 Nutrition, Dietetics and Sensory Sciences, Kansas State University, Manhattan, USA

**Keywords:** anti-inflammatory effects, bambara groundnut flour, diabetes management, low glycemic index, plant-based protein, sustainable crop, weight management

## Abstract

Bambara groundnut flour (BGNF), derived from *Vigna subterranea*, is a nutrient-dense, low-glycemic alternative to traditional flours, making it beneficial for diabetes and obesity management. Rich in protein (18-24%) and fiber (12-19%), it promotes satiety, regulates blood sugar, and supports muscle preservation during weight loss. Its complex carbohydrates and resistant starch allow for gradual glucose release, reducing blood sugar spikes in diabetics. BGNF also contains antioxidants and anti-inflammatory compounds that may help mitigate diabetes-related complications. Compared to almond, coconut, and chickpea flours, it offers a balanced nutrient profile suitable for glycemic control and weight management. However, challenges such as limited production, high costs, and low public awareness hinder its accessibility, particularly in the U.S. To fully realize its potential, research, including clinical trials, is needed to validate its health benefits, while efforts to scale production and raise awareness could help position BGNF as a sustainable, functional food for managing chronic health conditions.

## Introduction and background

Diabetes and obesity in the USA

The United States has witnessed a troubling rise in the prevalence of diabetes and obesity over the past few decades, becoming one of the most pressing public health concerns of our time. According to the Centers for Disease Control and Prevention (CDC), as of 2020, over 34.2 million adults in the U.S. had diabetes, which equates to around 10.5% of the population. Furthermore, an additional 88 million adults were estimated to have prediabetes, increasing their risk of developing type 2 diabetes in the future [1]. Alongside diabetes, obesity rates have surged to alarming levels, with more than 42.4% of U.S. adults classified as obese (BMI ≥ 30) and 9.2% affected by severe obesity (BMI ≥ 40) [2]. These conditions are closely interconnected, as obesity is a significant risk factor for developing type 2 diabetes [3].

The causes of this epidemic are multifaceted. Lifestyle factors such as poor diet and physical inactivity play a prominent role, alongside genetic predisposition and environmental influences [3,4]. One major driver is the "Western diet," characterized by high-calorie, nutrient-poor foods. This diet, which is rich in refined carbohydrates, saturated fats, and added sugars, contributes to excessive caloric intake, weight gain, and insulin resistance, key factors in the development of both obesity and type 2 diabetes [4-6].

The role of diet in managing diabetes and obesity

Dietary intervention is a cornerstone of managing both diabetes and obesity, as nutrition plays a central role in controlling blood glucose levels and body weight. For individuals with diabetes, managing blood sugar levels through diet is essential for preventing complications such as heart disease, neuropathy, and kidney damage. A key dietary approach for diabetes management involves regulating glycemic control, which refers to the maintenance of stable blood glucose levels. This is primarily achieved by limiting foods that cause rapid spikes in blood sugar, particularly refined carbohydrates and sugars, and instead focusing on low-glycemic index (GI) foods, which release glucose more slowly into the bloodstream [7,8]. Foods with a lower GI, such as legumes, whole grains, and fiber-rich vegetables, are recommended to improve insulin sensitivity and reduce the risk of hyperglycemia [9].

For individuals with obesity, dietary modifications that promote weight management are crucial. Even modest weight loss of 5-10% of body weight can lead to significant improvements in blood sugar control and overall metabolic health [10]. Diets rich in whole foods, including vegetables, fruits, lean proteins, and whole grains, along with reduced caloric intake, are effective in promoting weight loss and improving insulin sensitivity [11]. Additionally, increasing fiber intake is particularly beneficial, as fiber helps slow the absorption of sugars, supports digestion, and contributes to a feeling of fullness, which can aid in reducing overall caloric intake [12].

In recent years, there has been a growing interest in exploring alternative flours and plant-based foods as part of dietary interventions for diabetes and obesity. Traditional flours such as wheat, which are high in carbohydrates and low in fiber, may contribute to rapid blood sugar spikes. In contrast, alternative flours made from legumes or nuts, such as almond flour, coconut flour, and Bambara groundnut flour (BGNF), offer a more nutritionally dense option. These flours often have a lower glycemic index and higher fiber content, making them promising candidates for improving glycemic control and supporting weight management in diabetic and obese patients [13,14].

In summary, the rising prevalence of diabetes and obesity in the U.S. highlights the urgent need for effective dietary interventions. Given the central role of diet in managing both conditions, there is a growing interest in exploring new dietary options, such as BGNF, that offer the potential to better manage blood sugar levels and support healthy weight loss. The following sections will explore the nutritional composition and potential health benefits of BGNF as an alternative for diabetic and obese populations, particularly in the context of the U.S. market.

## Review

Bambara groundnut flour for health-conscious diets

In today’s health-driven society, there is an increasing demand for food products that not only support general well-being but also cater to specific dietary needs. One such product gaining attention for its impressive nutritional profile and functional properties is BGNF. Derived from the Bambara groundnut, an underutilized legume native to Africa, BGNF offers significant health benefits, particularly for individuals managing chronic conditions like diabetes and obesity.

The Bambara groundnut (Vigna subterranea) is a leguminous plant that thrives in poor soil conditions and drought-prone regions, making it an important crop for subsistence farming and food security in Africa [13]. Studies have highlighted its resilience and ability to grow in harsh climates with minimal resources, which positions it as a sustainable crop for addressing food insecurity [14,15]. Despite its environmental resilience and nutritional potential, the Bambara groundnut remains underutilized on the global market. This is particularly surprising given its rich nutrient profile, which makes it a valuable food source for health-conscious consumers [16].

BGNF, produced by milling the dried seeds of the Bambara plant, is rich in protein, dietary fiber, and a variety of essential minerals, including calcium, magnesium, and potassium. These nutrients are crucial for maintaining overall health, supporting muscle function, bone strength, and regulating blood pressure [13,16]. Studies comparing the nutritional profiles of Bambara groundnut and other legumes have also highlighted its superior mineral content, making it a promising alternative for addressing micronutrient deficiencies [14,17]. BGNF also contains a balanced mix of carbohydrates, making it suitable for individuals who need steady energy without blood sugar spikes, such as those managing diabetes [14].

One of the primary reasons why BGNF is gaining attention is its low GI. The GI of a food determines how quickly it raises blood sugar levels after consumption. Foods with a low GI release glucose gradually into the bloodstream, preventing the sharp spikes and crashes associated with high-GI foods like refined wheat flour. Research suggests that consuming low-GI foods like Bambara groundnut can significantly improve glycemic control and reduce insulin resistance, key factors in managing type 2 diabetes [8,9]. For diabetic patients, this gradual release is essential for maintaining stable blood sugar levels and reducing the risk of complications such as hyperglycemia [14]. Incorporating BGNF into their diets can, therefore, play a significant role in improving glycemic control and supporting overall metabolic health.

Nutritional and health benefits of Bambara groundnut flour

BGNF is particularly appealing due to its rich and varied nutritional content. One of its standout features is its high protein content, which is vital for muscle repair, immune function, and maintaining satiety, making it a great option for individuals who are seeking to control their weight. Studies show that BGNF can provide comparable, if not higher, protein levels than some commonly used legumes, such as chickpeas and lentils [17,18]. Additionally, its protein profile contains essential amino acids necessary for human health, including lysine and methionine, which are often limiting in other plant-based protein sources [19].

Dietary fiber is another significant component of BGNF. Fiber plays a crucial role in digestive health by promoting regular bowel movements and preventing constipation. Beyond this, the high fiber content in BGNF helps manage blood sugar levels by slowing the absorption of carbohydrates in the bloodstream [20]. For individuals managing obesity, dietary fiber promotes a feeling of fullness, which can reduce overall calorie intake and support weight management. Moreover, fiber-rich diets have been shown to significantly reduce risks of metabolic disorders, including type 2 diabetes and cardiovascular disease [21].

BGNF is also a rich source of essential minerals like calcium, magnesium, and potassium. Calcium is necessary for bone health and preventing conditions such as osteoporosis, especially in aging populations [22]. Magnesium and potassium, on the other hand, are essential for regulating blood pressure and maintaining heart health. Increased consumption of these minerals has been linked to lower rates of hypertension, making BGNF a highly nutritious option for individuals at risk of cardiovascular disease [23]. Moreover, antioxidants present in BGNF, such as polyphenols and flavonoids, are vital for combating oxidative stress. Oxidative stress occurs when there is an imbalance between free radicals and antioxidants in the body, often leading to chronic diseases like diabetes, obesity, and cardiovascular conditions. The antioxidants found in BGNF help neutralize harmful free radicals, protecting cells from damage and inflammation [24]. Emerging studies also suggest that Bambara groundnut polyphenols exhibit antimicrobial and anti-inflammatory properties, offering additional health benefits [23,25].

Bambara groundnut flour for diabetics and obese patients

For individuals managing diabetes, one of the most pressing dietary concerns is the need to avoid foods that cause rapid increases in blood sugar levels. With its low GI, BGNF is a powerful ally in controlling blood sugar. Unlike high-GI foods that lead to spikes in glucose levels, the carbohydrates in BGNF are absorbed slowly, allowing for better blood sugar regulation. Bambara groundnuts are a good source of complex carbohydrates, mostly oligosaccharides and polysaccharides. A significant portion of these complex carbohydrates is starch, potentially reaching up to 49.5% [7]. However, the actual starch content can vary considerably (22-49.5% of the dry weight) depending on factors like the plant's genetics, the environment it grows in, how mature the seeds are, and the specific testing methods used [14]. Because these complex carbohydrates are digested slowly, they help prevent blood sugar spikes after eating (postprandial hyperglycemia) and provide a sustained release of energy. This slow release can be especially helpful for people with diabetes, helping them avoid energy crashes [7,14]. Moreover, a study suggests that a low-GI diet, such as one incorporating BGNF, can improve insulin sensitivity and reduce the risk of long-term complications in individuals with diabetes [8].

Additionally, the fiber content of BGNF further aids in managing blood sugar levels. Fiber slows down the digestion of carbohydrates, leading to a more stable release of glucose into the bloodstream. This is particularly beneficial for people with type 2 diabetes, who need to manage insulin sensitivity and glucose fluctuations [16,21]. Research has also shown that dietary fiber helps lower hemoglobin A1C (HbA1c) levels, a critical indicator of long-term glycemic control in diabetic patients [5]. Integrating BGNF into a diabetic-friendly diet can therefore support long-term glycemic control and improve overall health outcomes.

For individuals with obesity, BGNF offers several key benefits. The high protein and fiber content of the flour promotes satiety, helping to reduce overall calorie intake [18]. Protein helps maintain muscle mass during weight loss, which is crucial for metabolic health, while fiber helps control hunger by providing a feeling of fullness for longer periods [20]. Moreover, studies indicate that fiber-rich foods can positively influence gut health, further aiding in weight management and reducing the risk of obesity-related metabolic disorders [8,9]. For obese individuals looking to manage their weight, BGNF can be a vital ingredient in maintaining a balanced, nutrient-dense diet.

Culinary versatility of Bambara groundnut flour

Beyond its impressive health benefits, BGNF also holds great promise in the kitchen. Like other alternative flours, BGNF can be used in a wide variety of recipes, from traditional dishes to innovative gluten-free baked goods. Its mild, slightly nutty flavor complements both sweet and savory dishes, allowing it to be used in everything from porridge and soups to cakes, muffins, and bread [26,27].

What sets BGNF apart is its ability to enhance the texture and nutritional value of food products, particularly in gluten-free baking, where maintaining the right texture can be challenging. The flour has excellent water absorption properties, which helps to create a moist and appealing texture in baked goods [28]. Incorporating Bambara groundnut flour into composite flours used for baking can improve protein and mineral content, making it a nutritionally superior alternative to conventional flours [29]. Moreover, its rich nutritional profile ensures that using BGNF in place of traditional flours enhances the health benefits of the dish without sacrificing flavor or texture [30].

A promising future for Bambara groundnut flour

Despite its significant benefits, BGNF remains underutilized in many parts of the world [13]. Its potential to promote sustainable agriculture, improve food security, and enhance health-conscious diets suggests that it deserves greater attention in both research and consumer markets. Studies have highlighted the Bambara groundnut’s resilience to drought and poor soils, making it an ideal crop for addressing food insecurity in regions facing climate challenges [31,32]. Its nutrient-dense composition and functional properties further position it as a valuable resource for combating malnutrition and supporting healthier diets [33].

With increased awareness and education, BGNF could emerge as a staple in the quest for healthier, more sustainable food options. Its use in everyday cooking and specialty diets, particularly for individuals managing diabetes and obesity, demonstrates its versatility and promise as a functional food ingredient [34]. Research suggests that investment in processing technologies and product development could further enhance the accessibility and appeal of Bambara groundnut-based products, increasing their integration into mainstream diets globally [35].

**Figure 1:**
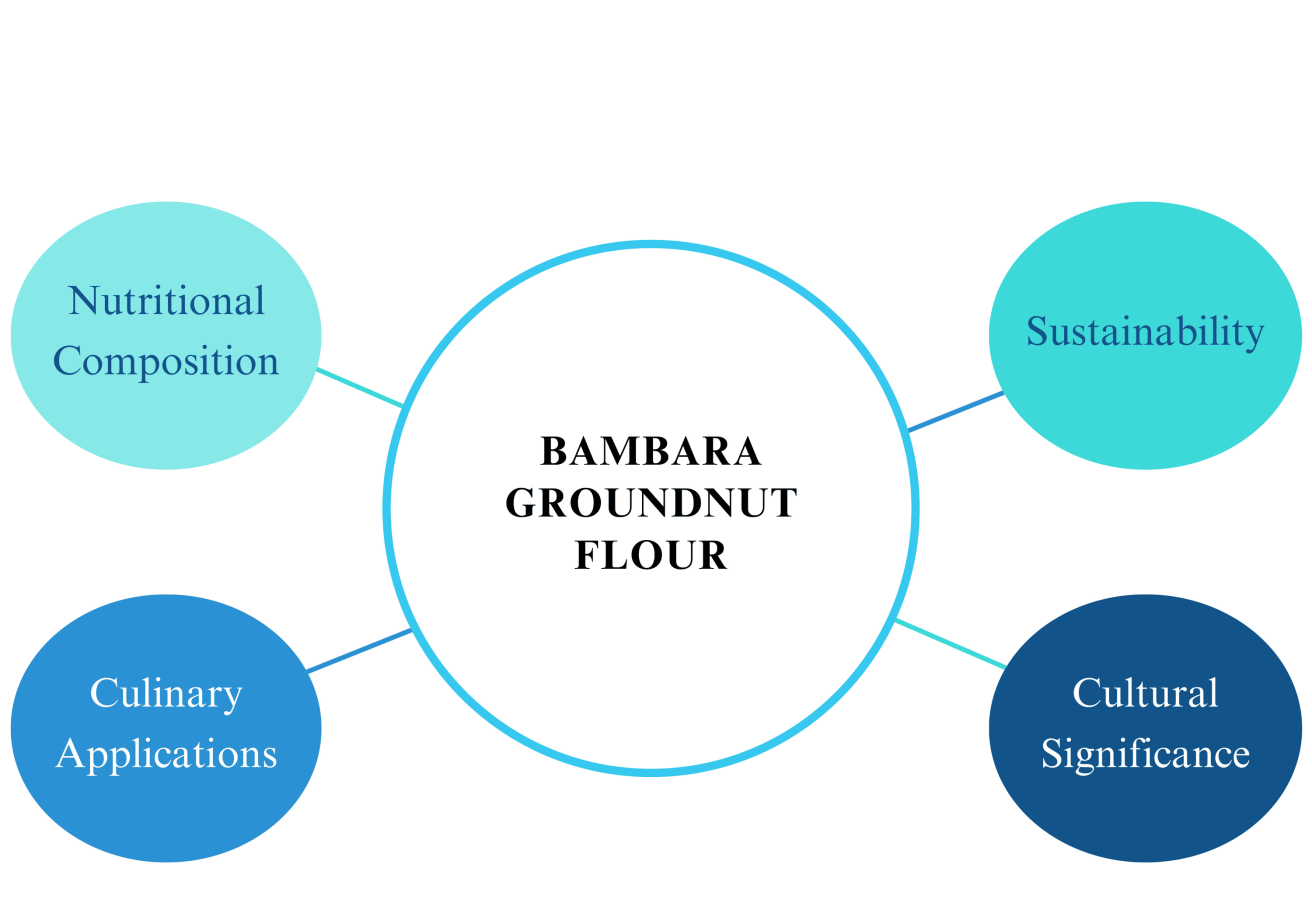
The Versatility of Bambara Groundnut Flour Image created by the author

BGNF is a nutritious and versatile food derived from a sustainable, drought-resistant crop that benefits soil health. It's used in diverse traditional and modern recipes, including gluten-free and plant-based options [34]. Promoting BGNF supports cultural food traditions, local farmers, and food security, particularly in African communities where it's a staple [14]. As depicted in Figure 1, BGNF’s advantages extend beyond its nutritional composition, which includes high protein, fiber, and essential micronutrients, to its role in promoting environmental sustainability through the cultivation of Bambara groundnut, a drought-tolerant and underutilized crop. Additionally, BGNF has diverse culinary applications, making it a versatile ingredient in various traditional and modern recipes. Its cultural significance as a staple food in many regions further underscores its importance in addressing food security and nutritional challenges globally.

Role of protein and fiber in diabetes and obesity management

The high protein and fiber content found in BGNF plays a pivotal role in managing chronic conditions such as diabetes and obesity. These macronutrients help regulate blood sugar levels and promote satiety, reducing overall caloric intake, which are key factors in controlling weight and managing blood glucose in diabetic individuals. Table 1 also highlights the nutritional composition of BGNF. 

**Table 1:** Nutritional Composition of Bambara Groundnut Flour (BGNF)

Nutrient	Composition	Health Benefits	References
Protein	18-24%	High-quality plant-based protein supports muscle repair, satiety, and metabolism. · Important for managing obesity and promoting satiety in weight management.	[16]
Dietary Fiber	12-19%	Aids in digestion and promotes gut health. Regulates blood sugar by slowing glucose absorption, beneficial for diabetic patients. · Promotes fullness, helping to control apetite.	[5]
Carbohydrates	50-60%	Complex carbs provide a slow release of glucose, ensuring steady energy. · Low glycemic index prevents blood sugar spikes, making it ideal for diabetics.	[14]
Fat	5-7%	Contains mostly unsaturated fats, beneficial for heart health. · Low-fat content supports healthy fat intake, especially for obese individuals.	[18]
Calcium	Moderate	· Vital for bone health and preventing osteoporosis, especially in older adults.	[16]
Magnesium	Moderate	Supports muscle function, blood pressure regulation, and insulin sensitivity. · Important for type 2 diabetes management	[9]
Potassium	Moderate	· Regulates blood pressure and supports nerve and muscle function.	[36]
Iron	Moderate	· Essential for oxygen transport and preventing anemia, particularly in at-risk populations.	[37]
Antioxidants (Polyphenols, Flavonoids)	High	Neutralizes free radicals, reducing oxidative stress linked to chronic diseases such as diabetes and obesity. · Reduces inflammation and promotes overall health.	[38]

Role of high-protein foods in satiety and weight management

Protein is one of the most satiating macronutrients, making high-protein foods an essential part of a diet aimed at reducing overall caloric intake and promoting weight management. BGNF, with its protein content ranging from 18% to 24%, is a plant-based protein source that provides these benefits. When consumed, proteins stimulate the release of hormones like cholecystokinin (CCK), glucagon-like peptide-1 (GLP-1), and peptide YY (PYY), which signal fullness to the brain, reducing feelings of hunger and promoting satiety for longer periods [39].

The thermic effect of food (TEF) also contributes to protein’s role in weight management. Protein has a higher TEF compared to carbohydrates and fats, meaning that the body uses more energy to digest and metabolize protein, increasing overall calorie expenditure. This makes protein an efficient nutrient for weight management and obesity prevention, as it helps maintain lean muscle mass while reducing fat stores [40]. For obese individuals, consuming high-protein foods like BGNF can be particularly beneficial as it helps curb overeating, lowering total calorie intake without triggering hunger or cravings [41].

Another important aspect is the muscle-preserving effect of protein. During periods of weight loss, there is often a risk of losing muscle mass along with fat. However, diets that are rich in protein can help preserve muscle tissue, which is critical for maintaining a healthy metabolism. Since muscle burns more calories at rest compared to fat, maintaining muscle mass is essential for long-term weight management [42]. BGNF provides the necessary protein to support these processes, making it a valuable addition to diets aimed at reducing body weight while preserving muscle mass.

Fiber-rich foods and glycemic control

The fiber content in BGNF, which ranges from 12% to 19%, plays a crucial role in controlling blood glucose levels, especially for individuals with diabetes. Soluble fiber forms a gel-like substance in the digestive system, slowing the digestion and absorption of carbohydrates. This delay reduces the rate at which glucose enters the bloodstream, resulting in lower post-meal blood sugar spikes [43].

For individuals with type 2 diabetes, this process is critical because it prevents sharp rises in blood sugar levels, which can lead to hyperglycemia. By consuming fiber-rich foods like BGNF, diabetics can achieve better glycemic control, reducing the strain on the pancreas to produce large amounts of insulin after meals. Over time, this improved glycemic control reduces the risk of complications such as cardiovascular disease and neuropathy [44].

Fiber also plays a significant role in improving insulin sensitivity, which is essential for managing diabetes and preventing its progression. High-fiber diets have been shown to enhance the body’s response to insulin, lowering insulin resistance-a condition often linked to type 2 diabetes and obesity [45]. Incorporating foods like BGNF, which are rich in fiber, helps improve insulin sensitivity and reduce the overall need for insulin, simplifying diabetes management.

In addition to its role in blood sugar regulation, fiber is also beneficial for weight management. High-fiber foods tend to promote satiety, reducing overall calorie consumption and helping individuals avoid overeating. Fiber adds bulk to the diet, which stimulates the digestive system and makes individuals feel full more quickly and for longer periods after meals [46]. For obese individuals, this property is particularly valuable as it supports long-term weight loss without excessive calorie intake.

Fiber-rich foods like BGNF also support digestive health by promoting regular bowel movements and nurturing a healthy gut microbiome. The gut microbiome is increasingly recognized as a key factor in metabolic health, with research showing that a diverse and balanced microbiome improves metabolism and reduces inflammation, both of which are essential for managing diabetes and obesity [47].

Glycemic index of Bambara groundnut flour

The GI and glycemic load (GL) are essential metrics in understanding how various carbohydrate-rich foods affect blood sugar levels. These measurements are particularly crucial for individuals managing diabetes and obesity, as they provide insights into how foods influence postprandial glucose responses and insulin levels. BGNF, with its low GI, offers a promising option for diabetic and obese patients who need to manage their blood sugar levels carefully.

The GI of a food is determined by how quickly it raises blood glucose levels after consumption compared to a reference food, such as pure glucose. Foods with a high GI cause a rapid increase in blood glucose, while low-GI foods release glucose slowly and steadily. BGNF has been said to have a low-GI range (typically between 41 and 51) depending on its processing methods and preparation [48]. These values indicate that BGNF causes a slow and gradual rise in blood glucose, making it an ideal carbohydrate source for individuals with diabetes who need to avoid rapid blood sugar spikes.

The low GI of BGNF can be attributed to its composition. BGNF contains a high percentage of complex carbohydrates and dietary fiber, both of which slow the digestion and absorption of carbohydrates. This ensures that glucose is released gradually into the bloodstream, thereby avoiding the sharp spikes in blood sugar that are commonly associated with higher-GI foods, such as refined wheat or rice flour [49]. For diabetic patients, this slower glucose release is beneficial for maintaining better glycemic control and avoiding post-meal hyperglycemia.

Comparison with other flours commonly used by diabetic and obese patients

When compared to other flours commonly consumed by diabetic and obese individuals, BGNF exhibits a considerably lower GI. For example, wheat flour, particularly refined wheat flour, has a GI ranging from 60 to 85, depending on its degree of processing [50]. This higher GI is due to the removal of the bran and germ during processing, which reduces the fiber content and allows carbohydrates to be digested more quickly. Foods made with refined wheat flour are thus rapidly absorbed, leading to significant increases in blood glucose levels after consumption.

Rice flour, commonly used in gluten-free products, also has a high GI, typically ranging from 70 to 95. The high GI of rice flour results from its lack of fiber and high content of rapidly digestible carbohydrates, leading to quick glucose absorption and subsequent blood sugar spikes [51]. In contrast, almond flour and coconut flour are recommended as low-GI alternatives, with almond flour having a GI under 40 [52]. However, BGNF offers a better macronutrient balance, providing significant protein and dietary fiber alongside its low glycemic impact, making it a more nutrient-dense choice.

Importance of low-GI foods for diabetes management

Low-GI foods are crucial in managing diabetes because they help regulate blood sugar levels more effectively than high-GI foods. Foods with a low GI lead to a slower rise in blood glucose levels after a meal, which helps prevent the dangerous blood sugar spikes that can occur after consuming high-GI foods. This steady release of glucose also reduces the insulin demand on the pancreas, which is especially important for individuals with type 2 diabetes, where insulin production may be impaired or insulin resistance is present [53].

For individuals with type 1 diabetes, where the body does not produce insulin, low-GI foods can help reduce the frequency of postprandial hyperglycemia and the amount of insulin needed to control blood sugar after meals. This, in turn, helps diabetic patients maintain more stable blood sugar levels throughout the day and reduces the risk of complications related to prolonged hyperglycemia, such as cardiovascular disease, nerve damage, and retinopathy [54].

Another important benefit of low-GI foods like BGNF is their ability to improve insulin sensitivity. Diets rich in low-GI foods are associated with improved insulin response and a lower risk of developing insulin resistance, a key factor in the development and progression of type 2 diabetes [55]. This makes BGNF an important dietary component for both preventing and managing diabetes.

Impact of glycemic load on obesity management

In addition to the GI, the concept of GL is also important when considering the effect of foods on blood sugar and insulin levels. GL takes into account both the GI of a food and the quantity of carbohydrates in a typical serving. A food may have a high GI, but if it contains only a small amount of carbohydrates per serving, it may have a relatively low GL and thus a smaller impact on blood sugar.

For individuals with obesity, low-GL foods are beneficial because they help manage appetite and prevent overeating. When high-GI foods are consumed, the rapid increase in blood glucose is often followed by a sharp decline, leading to hunger and cravings for more carbohydrate-rich foods [56]. This cycle of rapid glucose fluctuations can lead to excess caloric intake, making weight loss or maintenance more challenging. By choosing low-GL foods such as BGNF, individuals can experience more stable blood sugar levels, which helps control hunger and reduce the urge to overeat. Moreover, the fiber content in BGNF adds bulk to meals without adding excess calories, further aiding in weight management by promoting fullness and reducing overall calorie consumption [57].

Antioxidant and anti-inflammatory properties of Bambara groundnut flour

In addition to its beneficial effects on blood sugar regulation, BGNF offers potential advantages due to its antioxidant and anti-inflammatory properties. These properties are especially relevant for managing diabetes, as oxidative stress and chronic inflammation are closely associated with the progression and complications of the disease. The high content of natural antioxidants and anti-inflammatory compounds in Bambara groundnut may help mitigate the negative effects of oxidative stress and inflammation, providing further benefits for diabetic patients.

Oxidative stress occurs when there is an imbalance between the production of free radicals and the body's ability to neutralize them with antioxidants. In diabetes, chronic high blood sugar levels contribute to increased production of free radicals, leading to damage at the cellular level. This damage is particularly harmful to blood vessels, nerves, and organs, contributing to complications such as cardiovascular disease, retinopathy, and nephropathy [58]. A diet rich in antioxidants is essential to counteract these effects.

BGNF contains various antioxidant compounds, including polyphenols, flavonoids, and vitamin C, which are known for their ability to neutralize free radicals and reduce oxidative damage. Bambara groundnut exhibits significant antioxidant capacity, measured by its ability to scavenge free radicals [59]. These antioxidants protect cells from oxidative damage and may slow the progression of diabetes-related complications.

Research by Adedayo et al. revealed that the polyphenol content in Bambara groundnut significantly contributes to its antioxidant activity [60]. The study highlighted the potential of regular consumption of BGNF to reduce oxidative stress, thereby lowering the risk of complications such as diabetic neuropathy and cardiovascular disease. The antioxidant compounds in BGNF are primarily concentrated in its outer layers, suggesting that whole grain or minimally processed BGNF retains the highest levels of antioxidants. Processing methods like refining can reduce antioxidant content, emphasizing the importance of maintaining the integrity of BGNF for optimal health benefits.

Chronic inflammation is another critical factor in the development and progression of diabetes. Inflammatory responses in the body can exacerbate insulin resistance, impair glucose metabolism, and contribute to tissue and organ damage over time [61]. Managing inflammation is, therefore, an essential component of diabetes prevention and treatment.

BGNF contains various anti-inflammatory compounds, including flavonoids and phenolic acids, which have been shown to reduce markers of inflammation. A study by Udeh et al. identified significant anti-inflammatory effects in Bambara groundnut phenolic extracts, reducing levels of pro-inflammatory cytokines such as tumor necrosis factor-alpha (TNF-α) and interleukin-6 (IL-6) [59]. By lowering these inflammatory markers, the consumption of BGNF may reduce the overall inflammatory burden, improve insulin sensitivity, and minimize the risk of diabetes complications.

Resistant starch in BGNF also plays a role in inflammation reduction. Resistant starch undergoes fermentation in the large intestine, producing short-chain fatty acids (SCFAs) such as butyrate. SCFAs are known to exhibit anti-inflammatory effects by supporting gut health and reducing inflammation [62]. This link between gut health, inflammation, and metabolic health underscores another pathway through which BGNF contributes to diabetes management.

Research has highlighted the potential health benefits of Bambara groundnut (Vigna subterranea), particularly due to its rich phenolic content. A study by Samson et al. investigated the phenolic content and antioxidant properties of Bambara groundnut extracts, revealing significant antioxidant activity [63]. These findings suggest that the phenolic compounds present in Bambara groundnut may contribute to its potential anti-inflammatory effects. Further research is warranted to explore these properties and their implications for health.

The anti-inflammatory properties of BGNF are particularly important for diabetic patients. Reducing inflammation improves insulin sensitivity and glucose metabolism, helping to control blood sugar levels more effectively. These benefits can reduce the risk of developing complications such as cardiovascular disease, diabetic nephropathy, and neuropathy [61].

**Figure 2:**
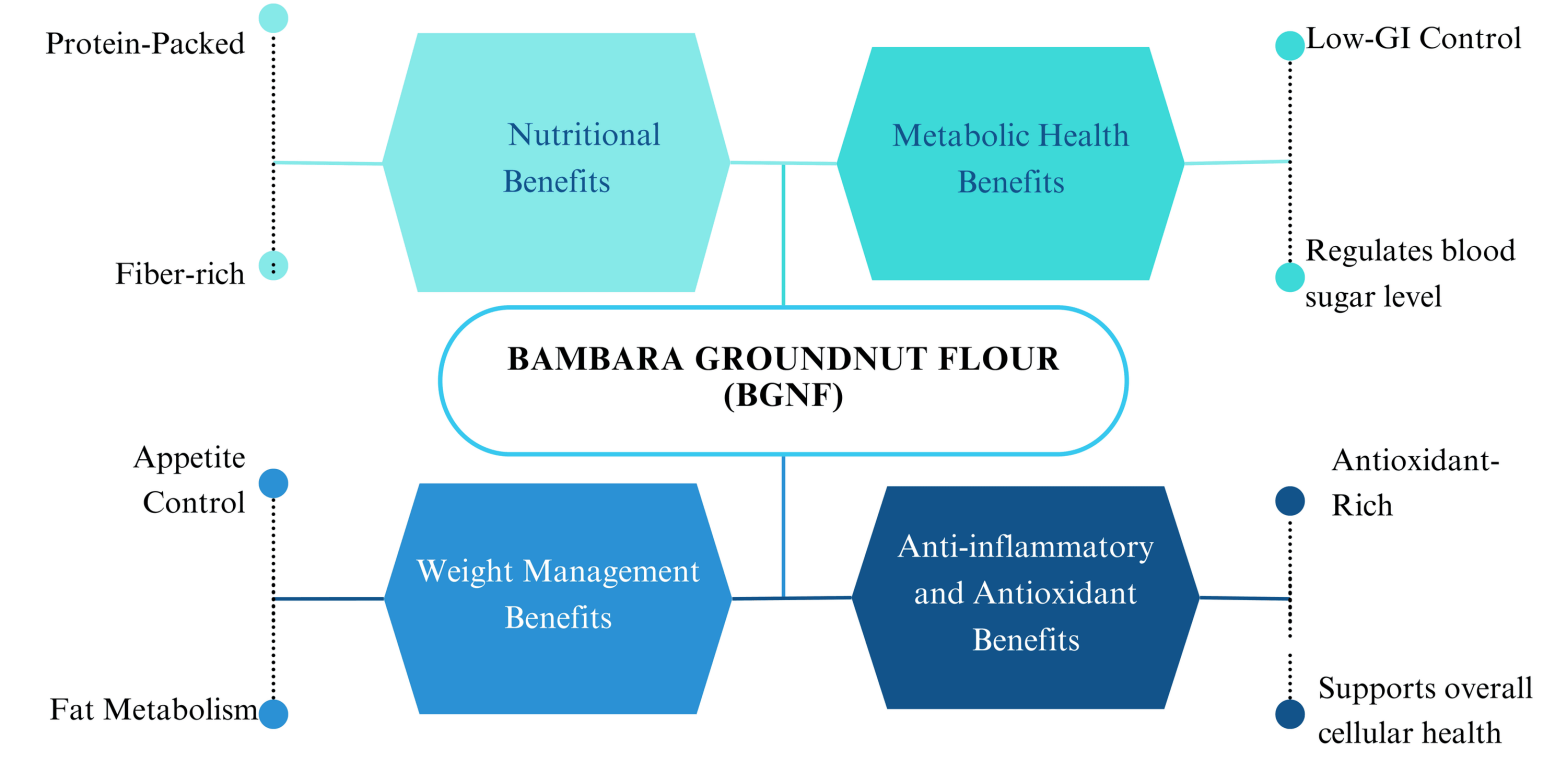
Some Health Benefits of Bambara Groundnut Flour Image created by the author

The multifaceted benefits of BGNF make it a valuable functional food for managing diabetes, obesity, and metabolic health. As illustrated in Figure 2, BGNF offers a range of nutritional, metabolic, and antioxidant benefits. Its high protein and fiber content contribute to appetite control, satiety, and fat metabolism, aiding weight management. Additionally, its GI helps regulate blood sugar levels, making it suitable for diabetic individuals. The flour's antioxidant properties, derived from polyphenols and flavonoids, further reduce inflammation and support overall cellular health. These combined properties position BGNF as a promising dietary intervention for improving metabolic and chronic health outcomes.

Impact on satiety and caloric intake

Satiety, or the feeling of fullness after eating, is a crucial factor in managing obesity and reducing caloric intake. One of the primary challenges in weight management is controlling hunger and preventing overeating, which can lead to excess calorie consumption. BGNF, with its high protein and fiber content, plays a significant role in promoting satiety and reducing overall caloric intake.

Studies indicate that high-protein and fiber-rich foods, such as Bambara groundnut, regulate appetite by stimulating satiety hormones. Protein increases the production of CCK, PYY, and GLP-1, which signal the brain to reduce hunger and prolong the feeling of fullness [39,40]. Additionally, protein’s thermic effect, which requires more energy to digest, contributes to increased calorie expenditure and further aids in weight management.

Dietary fiber also plays a pivotal role in satiety. Soluble fiber in BGNF forms a gel-like substance in the digestive tract, delaying digestion and absorption of nutrients, which extends the period of fullness after a meal [43]. A study by Jessie et al. found that individuals consuming legume-based flours, including BGNF, experienced reduced hunger and lower caloric intake compared to those consuming refined flour products [64].

Incorporating BGNF into the diet can help manage appetite, reduce overall calorie consumption, and support gradual and sustainable weight loss.

Comparison with other high-protein, low-GI foods

When compared to other high-protein and low-GI foods, BGNF offers a unique combination of nutrients. Almond flour, while low in carbohydrates and high in fat, contains less dietary fiber than BGNF, limiting its impact on appetite regulation [52]. Similarly, coconut flour is high in fiber but lacks the protein content required to preserve muscle mass during weight loss.

Chickpea flour, another legume-based alternative, shares similarities with BGNF in its high protein and fiber content. However, BGNF's resistant starch content gives it an added advantage in enhancing insulin sensitivity and fat metabolism, making it a stronger candidate for long-term weight management. As shown in Table 2, BGNF has a GI of 41-51, making it a moderate-GI option that provides gradual glucose release due to its high fiber (12-19%), resistant starch, and complex carbohydrates. These properties improve insulin sensitivity and make BGNF a balanced choice for glycemic control.

**Table 2:** Comparative Table: Diabetic-Friendly Flours

Flour Type	Glycemic Index (GI)	Key Nutrients	Glycemic Impact	Suitability for Diabetic Patients
Bambara Groundnut Flour	41-51	High protein (18-24%), fiber (12-19%), resistant starch, complex carbs	Moderate GI with gradual glucose release due to high fiber and resistant starch	Balanced option for glycemic control, moderate carbohydrate content, improves insulin sensitivity
Almond Flour	< 40	High fat and protein, low carbs	Very low GI, minimal blood sugar impact due to very low carbs	Ideal for low-carb and ketogenic diets, minimal glycemic response, best for those seeking very low carbohydrate intake
Coconut Flour	50-60	Extremely high fiber (35-45%), low carbs	Low GI, high fiber content slows carbohydrate digestion	Best for those needing very high fiberintake, low GI impact but lower in protein compared to BGNF
Chickpea Flour	45-60	High protein (20%), fiber (12-15%), complex carbs	Moderate GI, high fiber and protein reduce postprandial blood sugar spikes	Good option for balanced protein and fiber, but higher carb content compared to BGNF

Influence on body fat and weight loss

Beyond promoting satiety, BGNF may influence body fat reduction and overall weight loss. Its high protein content preserves lean muscle mass during weight loss, which is critical for maintaining a healthy metabolism. Protein-rich diets, such as those incorporating BGNF, help prevent the decline in resting metabolic rate often associated with muscle loss during calorie restriction [42].

The fiber and resistant starch content in BGNF contribute to fat metabolism. Resistant starch is fermented in the large intestine to produce short-chain fatty acids (SCFAs) like butyrate, which enhance fat oxidation and reduce fat accumulation [45]. A study by Chinma et al. showed that consumption of diets rich in legumes led to improved body composition, highlighting the potential of BGNF for promoting fat loss [65].

## Conclusions

BGNF is a nutrient-dense alternative for managing diabetes and obesity due to its low GI, high protein, and fiber content. Its gradual glucose release supports glycemic control, while fiber promotes satiety and protein aids muscle preservation during weight loss. Compared to other flours, BGNF’s balanced nutrient profile enhances insulin sensitivity and metabolic health.

To unlock its full potential, additional research, particularly clinical trials, is essential to validate its health benefits. Investments in production, supply chains, and public awareness campaigns will also be crucial for promoting BGNF as a functional food. With these efforts, BGNF could become a valuable tool in combating chronic health issues globally.
